# Vertical Segregation and Phylogenetic Characterization of Ammonia-Oxidizing Bacteria and Archaea in the Sediment of a Freshwater Aquaculture Pond

**DOI:** 10.3389/fmicb.2015.01539

**Published:** 2016-01-20

**Authors:** Shimin Lu, Xingguo Liu, Zhuojun Ma, Qigen Liu, Zongfan Wu, Xianlei Zeng, Xu Shi, Zhaojun Gu

**Affiliations:** ^1^Fishery Machinery and Instrument Research Institute, Chinese Academy of Fishery SciencesShanghai, China; ^2^College of Fisheries and Life, Shanghai Ocean UniversityShanghai, China; ^3^Chinese Academy of Fishery SciencesBeijing, China; ^4^Tongren Municipal Agricultural Commission (Government, Public)Tongren, China

**Keywords:** freshwater aquaculture pond, ammonia-oxidizing archaea, ammonia-oxidizing bacteria, sediment, depth distribution

## Abstract

Pond aquaculture is the major freshwater aquaculture method in China. Ammonia-oxidizing communities inhabiting pond sediments play an important role in controlling culture water quality. However, the distribution and activities of ammonia-oxidizing microbial communities along sediment profiles are poorly understood in this specific environment. Vertical variations in the abundance, transcription, potential ammonia oxidizing rate, and community composition of ammonia-oxidizing bacteria (AOB) and ammonia-oxidizing archaea (AOA) in sediment samples (0–50 cm depth) collected from a freshwater aquaculture pond were investigated. The concentrations of the AOA *amoA* gene were higher than those of the AOB by an order of magnitude, which suggested that AOA, as opposed to AOB, were the numerically predominant ammonia-oxidizing organisms in the surface sediment. This could be attributed to the fact that AOA are more resistant to low levels of dissolved oxygen. However, the concentrations of the AOB *amoA* mRNA were higher than those of the AOA by 2.5- to 39.9-fold in surface sediments (0–10 cm depth), which suggests that the oxidation of ammonia was mainly performed by AOB in the surface sediments, and by AOA in the deeper sediments, where only AOA could be detected. Clone libraries of AOA and AOB *amoA* sequences indicated that the diversity of AOA and AOB decreased with increasing depth. The AOB community consisted of two groups: the *Nitrosospira* and *Nitrosomonas* clusters, and *Nitrosomonas* were predominant in the freshwater pond sediment. All AOA *amoA* gene sequences in the 0–2 cm deep sediment were grouped into the *Nitrososphaera* cluster, while other AOA sequences in deeper sediments (10–15 and 20–25 cm depths) were grouped into the *Nitrosopumilus* cluster.

## Introduction

China is the world's largest producer, consumer, processor, and exporter of fish. China alone accounts for >60% of the global aquaculture volume and roughly half of the global aquaculture value (Cao et al., 2015). Currently, there are 2,623,180 ha of freshwater aquaculture ponds, and freshwater pond culturing is the major culture method in China (National Bureau of Statistics of China 2014, http://www.stats.gov.cn/english/statisticaldata/Quarterlydata/). To obtain more benefits from aquaculture, higher stocking densities are becoming prevalent. At the same time, large residual feed and feces are deposited into aquaculture sediments (Cao et al., 2015). A large amount of ammonia will be produced and released into the aquaculture water during the mineralization of organic matter. Ammonia not only significantly contributes to the eutrophication of aquaculture pond ecosystems, but is also one of the most toxic substances in intensive fish farming (Ackefors and Enell, 1994; Randall and Tsui, 2002). The high concentration of ammonia in aquaculture water has become a limitation for pond culturing in China.

Nitrification, the biological conversion of ammonia (NH_3_) to nitrate via nitrite (${\text{NO}}_{2}^{-}$), is a key process in nitrogen cycling in aquatic ecosystems (Merbt et al., 2012). Currently, the oxidation of NH_3_ to ${\text{NO}}_{2}^{-}$—the first and rate-limiting step of nitrification—is considered to be conducted by ammonia-oxidizing bacteria (AOB) and ammonia-oxidizing archaea (AOA; Koops and Pommerening-Röser, 2001). AOB fall into two phylogenetic lineages within the β- and γ-Proteobacteria (Kowalchuk and Stephen, 2001) and mainly belong to the genera *Nitrosomonas, Nitrosospira*, and *Nitrosococcus* (Kowalchuk and Stephen, 2001; Wang et al., 2014a). Based on genomic level comparisons, AOA were classified into a newly proposed branching phylum of the Archaea, named the Thaumarchaeota (Pester et al., 2011), and a recent study showed that AOA could be grouped into five major clusters: the *Nitrosopumilus* cluster (also called group I.1a AOA), the *Nitrosotalea* cluster (also called group I.1a-associated AOA), the *Nitrososphaera* cluster (also called group I.1b AOA), the *Nitrososphaera* sister cluster, and the *Nitrosocaldus* cluster [also called thermophilic AOA (ThAOA); Pester et al., 2012]. AOB and AOA both contain a homologous ammonia monooxygenase (AMO) that is responsible for catalyzing the first step in ammonia oxidation. The *amoA* gene, encoding the alpha subunit of AMO, has been used widely as a functional gene marker for tracking ammonia oxidizers in environmental samples (Rotthauwe et al., 1997; Francis et al., 2005). Thus far, many studies of AOA and AOB have been conducted; however, most of them have focused on large ecosystems, such as soils (Leininger et al., 2006; Tourna et al., 2011), oceans (Wuchter et al., 2006; Horak et al., 2013), lakes (Ye et al., 2009; Auguet et al., 2011, 2012; Zhao et al., 2013), and rivers (Jin et al., 2011; Sun et al., 2013; Wang et al., 2014b). There is a lack of studies of AOA and AOB in aquaculture ponds. Pond environments are smaller in area and shallower in depth, have limited water circulation, and are subject to large depositions of feeding debris. Moreover, the hypolimnion dissolved oxygen concentration was very low, although the super-saturation of oxygen usually occurs in the top layer during the daylight period (Chang and Ouyang, 1988). The trophic status and sediment properties make freshwater aquaculture ponds a good model system for studying the vertical distribution of AOA and AOB.

The oxidation of ammonium mainly occurs in the pond sediments, probably because of photoinhibition, and we previously found a low abundance of ammonia-oxidizing microorganisms in freshwater aquaculture water throughout the year (Lu et al., 2015). Hence, the aims of the present study were to investigate the activity and biodiversity in different sediment layers of a selected freshwater aquaculture pond in East China, and to quantitatively assess its AOA and AOB. In order to better understand the vertical distribution of AOA and AOB, we also partially characterized the physical and chemical factors [pH, dissolved oxygen (DO), total organic carbon (TOC), ammonium (${\text{NH}}_{4}^{+}$) and ${\text{NO}}_{2}^{-}$] of the sediment.

## Materials and methods

### Sediment samples and background

Samples were collected from a freshwater aquaculture pond located at the Research Center for Pond Ecosystem Engineering, Chinese Academy of Fishery Sciences [30°56′ N, 121°09′ E], Shanghai, China. The sampling pond had a surface area of ~5000 m^2^ and an average depth of about 1.6 m. Wuchang bream (*Megalobrama amblycephala*), grass carp (*Ctenopharyngodon idella*), silver carp (*Hypophthalmichthys molitrix*), and bighead carp (*Hypophthalmichthys nobilis*) were raised in the pond for commercial use from 2008 to 2011 and 2014 to 2015, and the production of fish was about 1200 kg km^−2^ per year. From 2012 to 2013, the submerged plant *Chara fragilis Desv*. was widely cultivated, and crab, whose production was about 150 kg km^−2^ per year, were raised in the sampled pond for commercial use. The sampled pond was dry during winter.

Three sediment cores (5 cm diameter and 50 cm depths) were collected from the aquaculture pond in October 2014 using a polyvinylchloride pipe. Then, the sediment cores were placed in sterile plastic bags, sealed, and transported to the laboratory on ice. Later, they were sectioned to 2 cm from 0 to 10 cm depths, and to 5 cm at 10–50 cm depths, and then we mixed the different cores from each sample for each depth. One portion was incubated to determine the ammonia oxidation activities immediately after arrival, another portion was used for an analysis of chemical components, and subsamples were stored at −80°C for subsequent DNA and RNA extractions and molecular analysis.

### Chemical analytical procedures of sediments

Ammonium (${\text{NH}}_{4}^{+}-$N) and nitrite (${\text{NO}}_{2}^{-}-$N) were extracted from the sediments with 2 M KCl and measured photometrically using Nessler's reagent, and spectrophotometrically using N-(1-Naphthyl)-ethylenediamine dihydrochloride, respectively (Hou et al., 2003; Lu et al., 2015). The pH of sediment was determined after mixing it with water at a ratio (sediment/water) of 1:2.5, and sediment organic matter was determined using a total carbon analyzer (Vario TOC, Elementar, Germany; Zhu et al., 2011). In July 2015, sediment samples were collected in the same pond as previously described, and the DO concentration in fresh sediments was measured immediately on a fishing boat using an OXY Meter S/N 5015 with an microelectrode sensor (OX-50 μm, Unisense, Aarhus, Denmark), as described by Gundersen et al. (1998).

### Measuring the potential ammonia oxidation rate

Potential ammonia oxidation rates were measured using the chlorate inhibition method (Kurola et al., 2005). Briefly, 5.0 g of fresh sediment was added to 50 ml centrifuge tubes containing 20 ml of phosphate buffer solution (NaCl, 8.0; KCl, 0.2; Na_2_HPO_4_, 0.2; NaH_2_PO_4_, 0.2 g L^−1^) containing 1 mM (NH_4_)_2_SO_4_. Potassium chlorate was added to the tubes to a final concentration of 10 mM to inhibit nitrite oxidation. The suspension was incubated with shaking (300 rpm) for 0.5 h at 25°C in the dark; then, the suspension was incubated without shaking for 24 h at 25°C in the dark; afterwards, nitrite was extracted with 5 ml of 2 M KCl and determined spectrophotometrically at 540 nm using N-(1-Naphthyl) ethylenediamine dihydrochloride. The potential ammonia oxidation rates were calculated based on the change in the nitrite concentrations.

### Nucleic acid extraction, quantitative polymerase chain reaction (qPCR), and reverse transcription

Extraction of DNA from the sediment samples was conducted, and two controls were performed to estimate the possible inhibition of qPCR performance by the co-extracted polyphenolic compounds or humic acids in the sediment, as described by Lu et al. (2015). Total RNA was extracted from the sediment samples using the E.Z.N.A.® Soil RNA Mini Kit (Omega Bio-Tek, Norcross, GA, USA) according to the manufacturer's instructions. RNA was reverse transcribed into cDNA using the PrimeScript RT Master Mix (Perfect Real Time; TaKaRa Biotechnology Dalian Co., Ltd., Dalian, China). Absence of contamination from DNA and chemical reagents was verified by performing the same reactions without reverse transcriptase or template, respectively. The obtained cDNAs were stored at −80°C for further analysis. qPCR was used to estimate the abundance of ammonia-oxidizing microorganisms' *amoA* mRNA and DNA, as well as total bacterial and Crenarchaeota 16S rRNA genes. qPCR was performed using a SLAN real-time PCR detection system (Hongshi Medical Technology Co. Ltd., Shanghai, China). The primers and reaction conditions for qPCR are listed in Table 1. qPCRs were conducted in a total volume of 20 μL containing 10 μL of SYBR Premix Ex Taq II for the AOB *amoA* gene or SYBR Premix Ex Taq for the AOA *amoA* gene and total bacterial and Crenarchaeota 16S rRNA genes (Takara), 1 μL of DNA template, and 0.2 mg mL^−1^ bovine serum albumin (BSA). A negative control without DNA template was subjected to the same procedures to exclude or detect any possible contamination. After qPCR, the specificity of the amplification was verified by a melting curve analysis and agarose gel electrophoresis. All measurements were performed in triplicate.

**Table 1:** **Primers used for PCR amplification for library construction and real-time PCR quantification**.

**Target gene**	**Primer**	**Sequence (5′–3′)**	**Concentration (nM)**	**Condition**	**References**
AOA *amoA*	Arch-amoAF	STAATGGTCTGGCTTAGACG	200	95°C for 30 s; 35 cycles of 95°C for 5 s, 53°C	Francis et al., 2005
	Arch-amoAR	GCGGCCATCCATCTGTATGT	200	for 1 min, 72°C for 70 s, and 80°C for 20 s (read plant);	
β-AOB *amoA*	*amoA*-1F	GGGGTTTCTACTGGTGGT	200	95°C for 30 s; 40 cycles of 95°C for 5 s, 54°C	Rotthauwe et al., 1997
	*amoA*-2R	CCCCTCKGSAAAGCCTTCTTC	200	for 40 s, 72°C for 70 s, and 80 for 20 s (read plant);	
Bacteria	1055f	ATGGCTGTCGTCAGCT	400	95°C for 30 s; 35 cycles of 95°C	Amann et al., 1995
16S rRNA	1392r	ACGGGCGGTGTGTAC	400	for 5 s, 54°C for 45 s, 72°C for 45 s (read plant);	Wilson et al., 1990
Crenarchaeota	771F	ACGGTGAGGGATGAAAGCT	400	95°C for 30 s; 40 cycles of 95°C for 5 s, 54°C	Torsten et al., 2003
16S rRNA	957R	CGGCGTTGACTCCAATTG	400	for 45 s, 72°C for 40 s, and 80°C for 20 s (read plant);	

Standard curves for qPCR were developed as described previously (Lu et al., 2015). External standard curves ranging from 10^1^ to 10^5^ copies per microliter of the archaeal and bacterial *amoA* genes or 10^4^ to 10^8^ copies of the bacterial and Crenarchaeota 16S rRNA genes were generated during the process of qPCR. Standard curve coefficients of variation and efficiencies were as follows: AOA (*R*^2^ = 0.999, efficiency = 94.4%), AOB (*R*^2^ = 0.999, efficiency = 90.9%), bacterial 16S rRNA (*R*^2^ = 0.998, efficiency = 90.0%) and crenarchaeota 16S rRNA gene (*R*^2^ = 0.999, efficiency = 82.7%). The results of the real-time PCR were expressed as the number of *amoA* or 16S rRNA gene copies g^−1^ of sediment (dry weight).

### Cloning, sequencing, and phylogenetic analysis

The purified PCR products were ligated and cloned using the pMD ™18-T Vector (Takara). In total, 105 and 76 clones of the AOA and AOB *amoA* gene PCR products, respectively, were successfully picked and sequenced. Operational taxonomic units (OTUs) were defined as sequence groups in which sequences differed by ≤2% for AOA and ≤3% for AOB. Neighbor-joining phylogenetic trees were constructed using MEGA 5.05 (Kumar et al., 2008).

### Statistical analysis

The estimated coverage of the constructed *amoA* gene libraries was calculated as *C* = [1−(*n*∕*N*)] × 100%, where *n* is the number of unique OTUs and *N* is the total number of all clones in a library. Indices of the *amoA* genotype diversity (Shannon–Wiener, H), richness estimations (Chao index), and rarefaction analysis were calculated using DOTUR (Schloss and Handelsman, 2005). Correlations between AOA abundance and environmental factors and One-way analysis of variance (ANOVA) were analyzed using SPSS 16.0 software.

### Nucleotide sequence accession numbers

The nucleotide sequences obtained in this study were deposited in the GenBank database under accession nos. KR081161–KR081236 for AOB and KR081056–KR081160 for AOA.

## Results

### Abundances, ammonia oxidation rates, and expression of AOA and AOB

**Figure 1:**
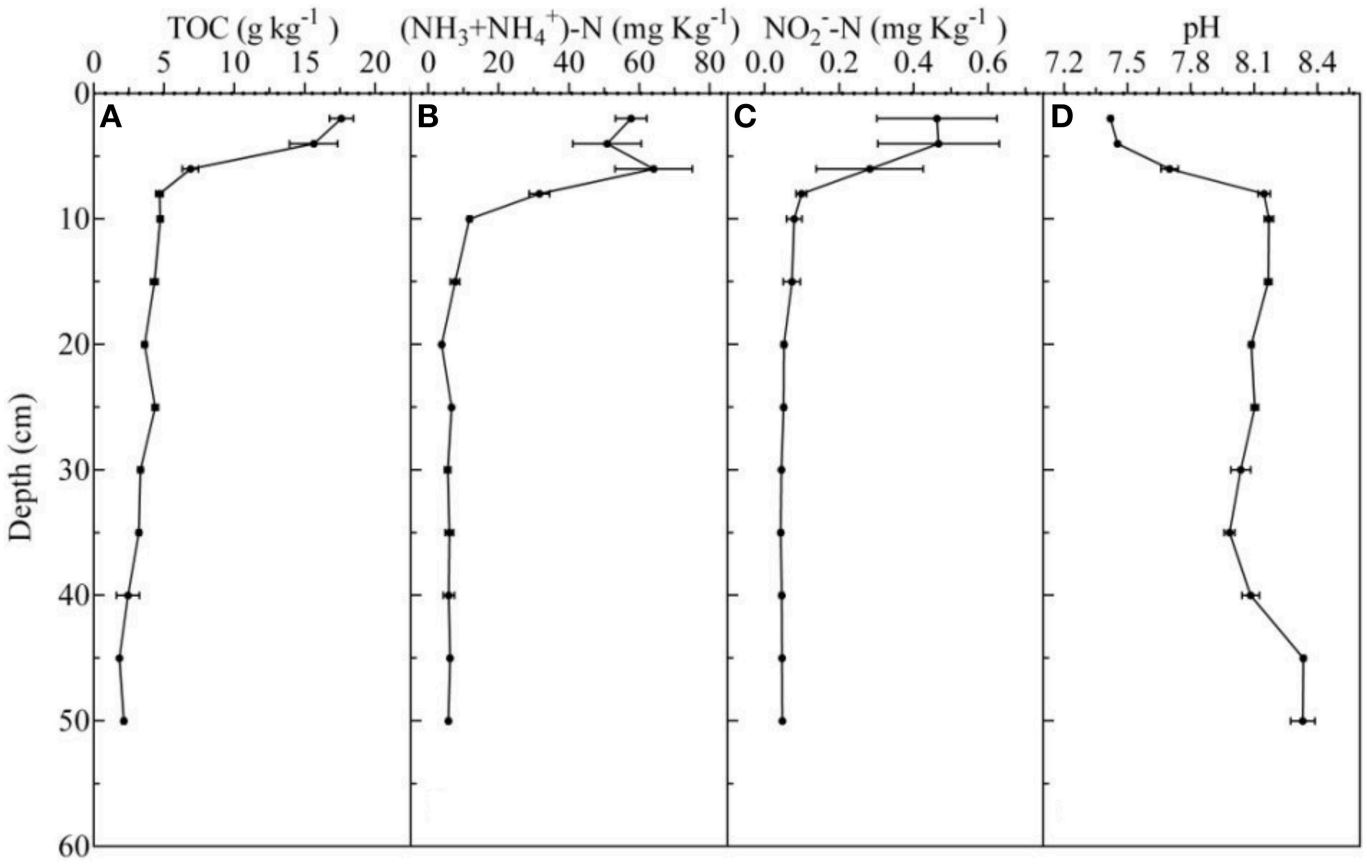
**Vertical distribution of total organic matter (A), ammonia nitrogen (B), nitrite nitrogen (C), pH (D), which was detected in October 2014**.

The vertical distribution profiles of TOC, ${\text{NH}}_{4}^{+}-$N, ${\text{NO}}_{2}^{-}-$N, and pH in every sediment core are shown in Figure 1. Briefly, the TOC, ${\text{NH}}_{4}^{+}-$N, and ${\text{NO}}_{2}^{-}-$N concentrations were high in 0–6 cm sediments, and decreased rapidly from 6 to 10 cm.

**Figure 2:**
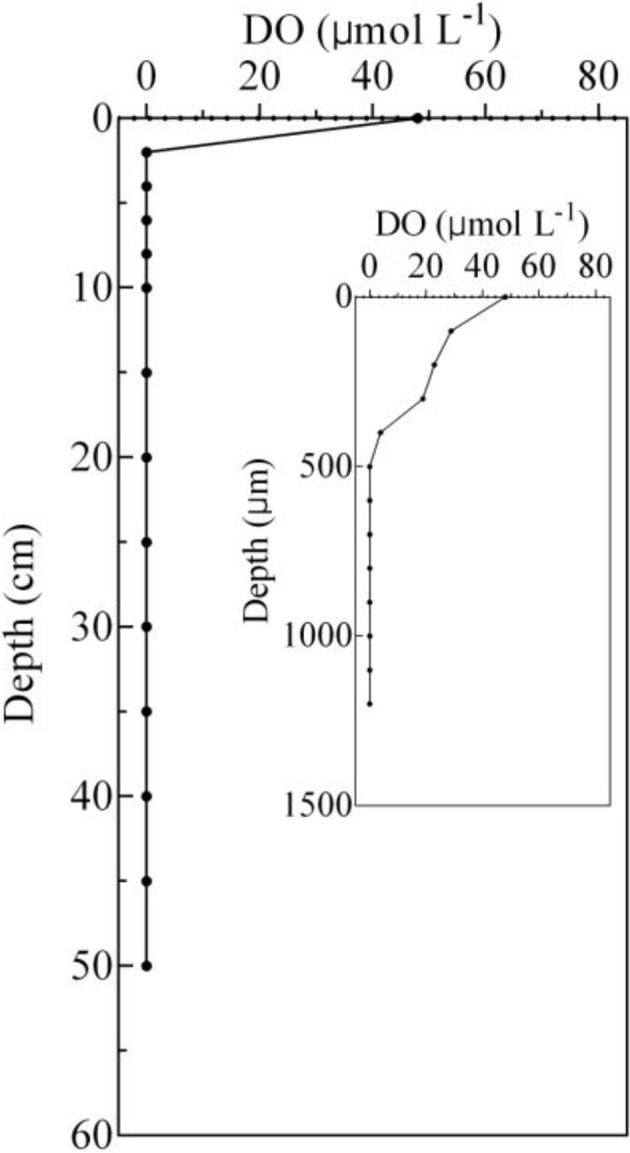
**Vertical distribution of DO in the sediment cores, which was detected in July 2015**.

The concentrations of TOC, ${\text{NH}}_{4}^{+}-$N, and ${\text{NO}}_{2}^{-}-$N ranged from 1.81 ± 0.04 to 17.60 ± 0.85 g kg^−1^, 3.88 ± 0.45 to 64.09 ± 11.01 mg kg^−1^, and 0.05 ± 0.01 to 0.47 ± 0.16 mg kg^−1^, respectively. The pH ranged from 7.42 to 8.33, and the lowest and the highest value occurred at the 0–2 and 45–50 cm, respectively. The depth of the DO detection limit was 500 μm, and the DO concentrations ranged from 0 to 48.01 μmol L^−1^ (Figure 2).

**Figure 3:**
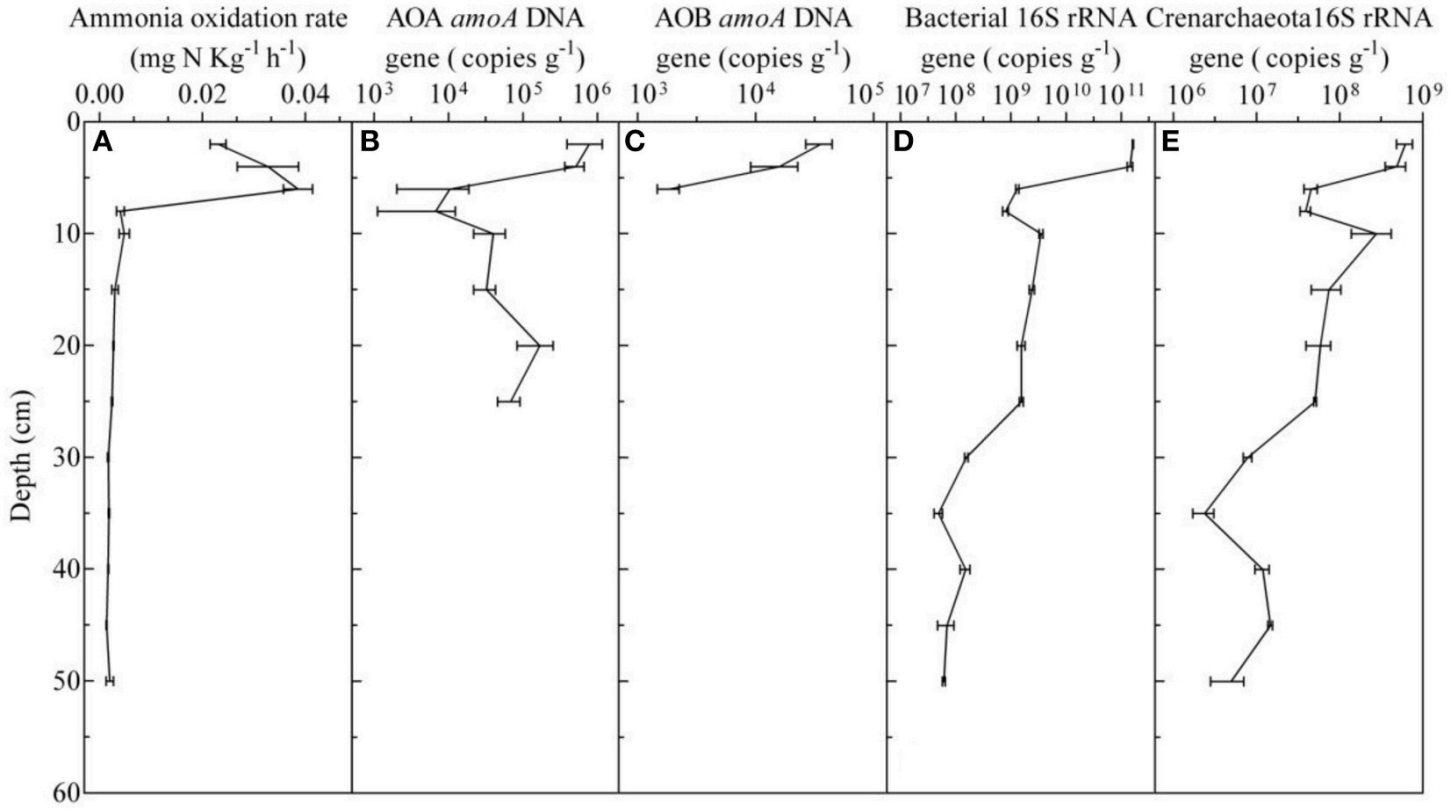
**Vertical distribution of ammonia oxidation activity (A), AOA ***amoA*** gene (B), AOB ***amoA*** gene (C), and bacterial (D) and crenarchaeota (E) 16S rRNA gene copy numbers in the sediment cores, which was detected in October 2014**.

To detect the presence of AOA, AOB, as well as the Crenarchaeota and total bacterial, the *amoA* and 16S rRNA genes from sediment core samples were amplified. The results showed that the depth limits for detecting the AOA and AOB *amoA* genes were 25 and 6 cm, respectively, and that the concentrations of the AOA *amoA* gene (ranging from 6.82 ± 2.28 × 10^4^ to 7.79 ± 3.88 × 10^5^) were higher than those of the AOB (1.88 ± 0.39 × 10^3^ to 3.60 ± 0.91 × 10^4^) by an order of magnitude, which suggested that the AOA, as opposed to the AOB, were the numerically predominant ammonia-oxidizing organisms in the surface sediment (Figure 3). Additionally, a positive PCR product was obtained for the Crenarchaeota and total bacteria in every sample, and the 16S rRNA concentrations ranged from 4.94 ± 2.12 × 10^6^ to 6.18 ± 1.33 × 10^8^, and 6.10 ± 0.36 × 10^7^ to 1.62 ± 0.04 × 10^11^ copies g^−1^, respectively (Figure 3). Linear relationships between different environmental factors and the *amoA* gene abundance of the AOA and AOB were characterized using Pearson's correlation coefficient. It was found that the abundance of AOA and AOB positively correlated with the TOC in the sediments (*R*^2^ = 0.838, *P* < 0.01; *R*^2^ = 0.852, *P* < 0.01), while the AOA and AOB abundances were negatively correlated with pH (*R*^2^ = −0.755, *P* < 0.01; *R*^2^ = −0.787, *P* < 0.05, respectively). No significant correlations were detected between the AOA and AOB abundances and the concentrations of ${\text{NH}}_{4}^{+}-$N and ${\text{NO}}_{2}^{-}-$N in sediments.

**Figure 4:**
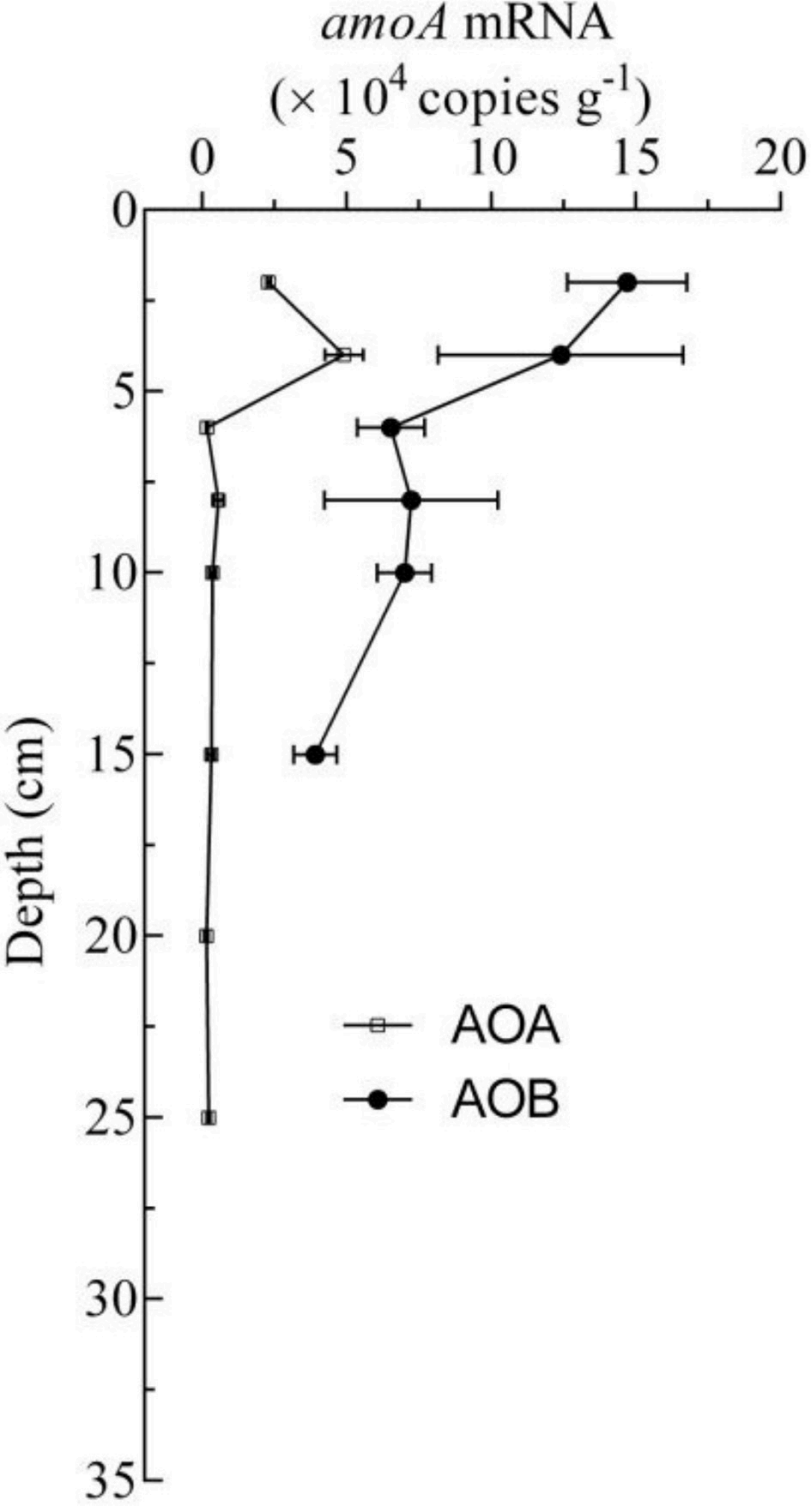
**Vertical distribution of transcripts of AOA and AOB ***amoA*** mRNA copy numbers in the sediment cores**.

To obtain more detailed information about the AOA and AOB in the freshwater aquaculture pond sediments, the potential ammonia oxidation rate was obtained from every sample, and it ranged from 0.0014 ± 0.0001 to 0.0386 ± 0.0028 mg kg^−1^ h^−1^. The potential ammonia oxidation rates in 0–6 cm deep sediments were significantly higher than those in other sediment layers (*P* < 0.01; One-way ANOVA; Figure 3A). The expression of the AOA and AOB *amoA* genes was calculated using the abundance of the PCR products that were amplified from cDNAs. Despite the fact that the depth of the AOB detection limit was 6 cm, AOB *amoA* gene expression could be detected at 8–10 cm in sediment cores, and it was higher than that of AOA in 0–10 cm depths by 2.5–39.9-fold (Figure 4).

### Diversity of AOA and AOB

**Figure 5:**
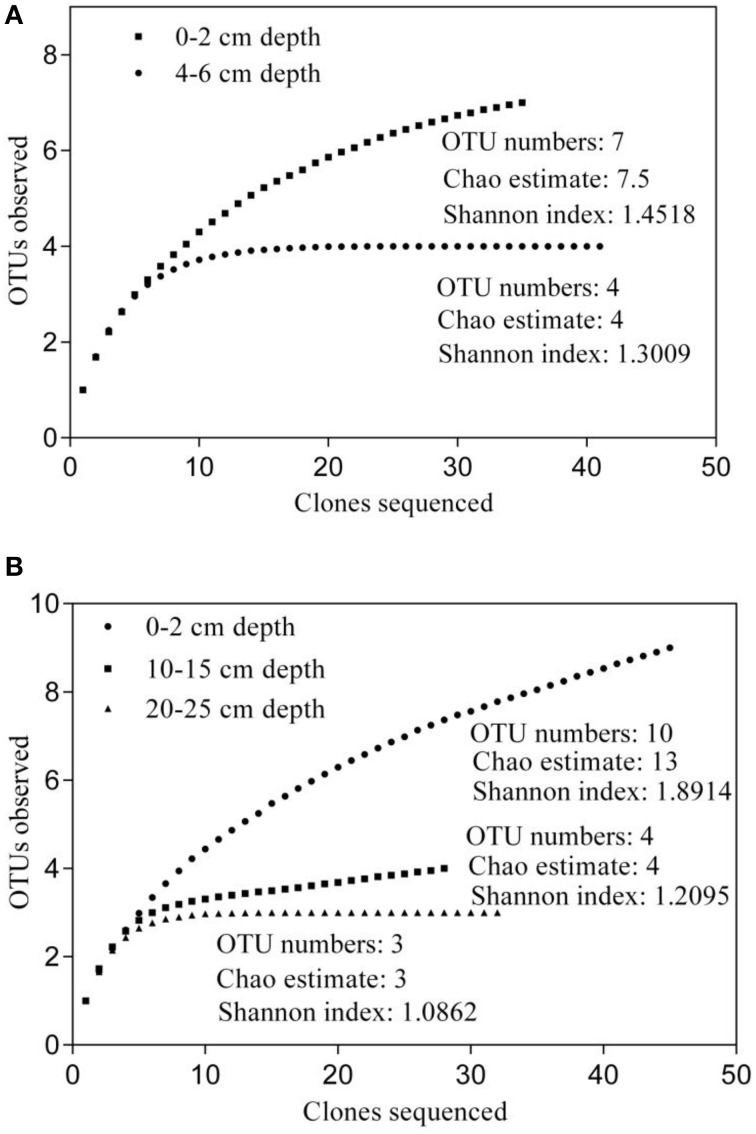
**Rarefaction analysis of ammonia-oxidizing bacterial (AOB) (A) and ammonia-oxidizing archaea (AOA) (B) communities in freshwater pond sediment**. The DOTUR program was used with 3% sequence variation for AOB, or with 2% sequence variation for AOA, for the operation taxonomic unit (OTU) determination.

**Figure 6:**
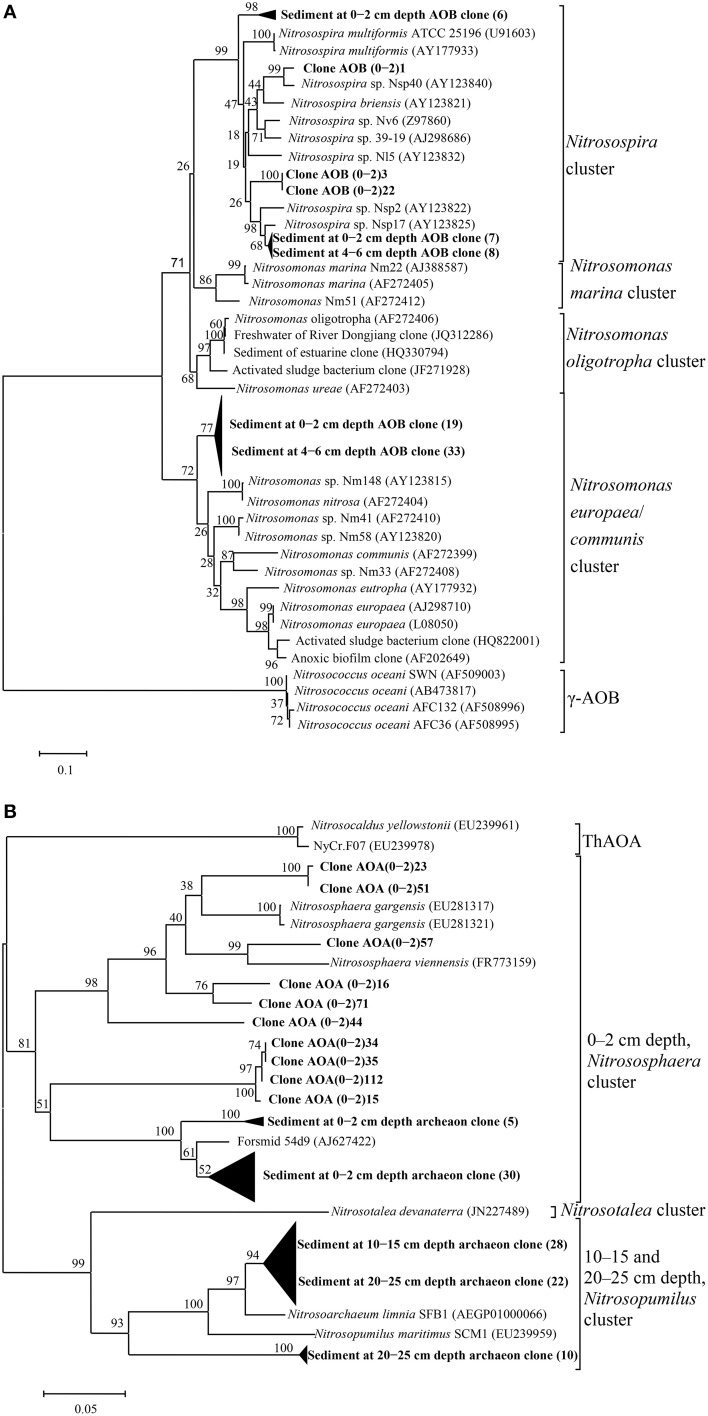
**Phylogenetic tree of AOB (A) and AOA (B) based on partial ***amoA*** gene sequence (472 bp for AOB and 597 bp for AOA) from the aquaculture pond sediment and some related sequences from GenBank reconstructed via the neighbor-joining method by fixing the Kimura 2-parameter evolution model with a bootstrap level of 1000 replications in MEGA 5.0**. The numbers close to the nodes represent the bootstrap values (*n* = 1000 replicates); Scale bar represents 0.1 (for AOB) or 0.05 (for AOA) nucleic acid substitutions per nucleotide position; Clones obtained from this experiment are highlighted in bold and are designated by sample names; the numbers in brackets represent the number of clones.

To investigate the diversity and community composition of ammonia-oxidizing populations, sediment layers with depths of 0–2 and 4–6 cm, as well as 0–2, 10–15 cm, and 20–25 cm, were selected for the construction of clone libraries of bacterial and archaeal *amoA* genes, respectively. Five clone libraries of the *amoA* gene were constructed to explore the diversity of AOB and AOA. The estimated coverage (C) of the five clone libraries ranged from 91 to 100%, which, together with the rarefaction analysis (Figure 5), indicated that the bacterial and archaeal *amoA* genotypes in the sediments could be well-represented by these clone libraries. As shown in Figure 5A, the OTU numbers, Chao estimate, and Shannon index of AOB in the 4–6 cm deep sediment were all less than those of AOB at a 0–2 cm depth, which indicated that the diversity of AOB decreased with increasing sediment depth. A phylogenetic analysis of bacterial *amoA* sequences suggested that the AOB community in aquaculture pond sediments consisted of two groups: the *Nitrosospira* and *Nitrosomonas* clusters (Figure 6A). The sequences related to *Nitrosomonas* spp. were predominant over those of *Nitrosospira* in AOB communities in the freshwater pond sediment.

An obvious variation in the AOA community and structure with sediment depth was also observed. As shown in Figure 5B, the diversity of AOA decreased with increasing sediment depth. All the AOA *amoA* gene sequences in 0–2 cm deep sediments were grouped into the *Nitrososphaera* cluster, while all the AOA sequences in 10–15 cm deep sediments, as well as a portion of the AOA sequences in the 20–25 deep sediments were grouped into a branch that belongs to the *Nitrosopumilus* cluster; the other AOA sequences in the 20–25 cm deep sediments were grouped into another branch of the *Nitrosopumilus* cluster. No sequences belonging to the ThAOA and *Nitrosotalea* clusters were detected (Figure 6B).

## Discussion

### Abundances, ammonia oxidation rates, and expression of AOA and AOB

A significant positive correlation between the abundance of AOA and TOC was observed. This may indicate that AOA are able to assimilate organic substrates and thereby be able to grow mixotrophically or even heterotrophically. This view is supported by studies of archaeal isolates from soil and marine sediments (Tourna et al., 2011; Qin et al., 2014), although our results are quite different from those that showed a negative correlation between AOA abundance and TOC concentrations in the sediments of a eutrophic lake and river (Wu et al., 2010; Wang et al., 2014b).

Because of large depositions of feeding debris and feces in the aquaculture pond, the surface sediments were rich in organic substances and exhibited a high ${\text{NH}}_{4}^{+}-$N concentration (Figure 1). The abundance of the AOA *amoA* gene was one order of magnitude higher than that of the AOB in the surface sediment (0–6 cm depth), which suggested that AOA, as opposed to AOB, were the numerically predominant ammonia-oxidizing organisms in the surface sediment of the freshwater aquaculture pond. We observed the same phenomenon in 10 other Chinese freshwater pond sediments (Lu et al., 2015). Our result contradicts the concept that AOA prefer lower ${\text{NH}}_{4}^{+}$ concentration environments because of their higher specific affinity for ${\text{NH}}_{4}^{+}$, whereas AOB prefer higher ${\text{NH}}_{4}^{+}$ concentrations (Martens-Habbena et al., 2009; Habteselassie et al., 2013).

AOA, rather than AOB, were the numerically predominant ammonia-oxidizing organisms in the surface sediment. This could be attributed to the fact that AOA are more resistant to low levels of DO (Coolen et al., 2007; Molina et al., 2010; Bouskill et al., 2012). The oxygen dynamics in aquaculture ponds differ from those of other aquatic systems, as pond environments are smaller, have limited water circulation, and are subjected to large deposits of feeding debris. The hypolimnion DO concentration was rarely >62.5 μ mol L^−1^ in an aquaculture pond, although the super-saturation of oxygen usually occurs during the daylight period (Chang and Ouyang, 1988). Moreover, for example, the DO concentration at the water-sediment interface was only 48.1 μ mol L^−1^ during another season (July 2015), and it reached zero when at depths >500 μm.

Apart from the ${\text{NH}}_{4}^{+}-$N and TOC concentrations, pH has been suggested to be an important environmental factor that influences the distribution of AOB and AOA (He et al., 2007; Yao et al., 2011; Hu et al., 2014; Jiang et al., 2015). In this study, a significant negative correlation was found between pH and the abundance of the AOA *amoA* gene, indicating that the number of AOA decreased with decreasing pH-values. This finding is consistent with the physiological features of isolated AOA strains (Jong-Geol et al., 2012; Qin et al., 2014) and previous studies conducted in soil (Nicol et al., 2008; Hu et al., 2014). This effect might be associated with the reported requirement for the use of NH_3_, not ${\text{NH}}_{4}^{+}$ (Martens-Habbena et al., 2009). The sediment pH was significantly negatively correlated with the abundance of the AOB *amoA* gene, indicating that pH was an important factor that controlled the AOB abundance in aquaculture pond sediment. This finding is consistent with the result obtained from an alkaline sandy loam (Shen et al., 2008). A study of an AOB isolate from freshwater showed that it could grow in a wide pH range, although the highest growth rate occurred at pH 7–7.5 (Elizabeth et al., 2012). In this study, the pH in the surface (0-6 cm depth) sediment ranged from 7.4 to 7.7. Although the pH only increased by 0.3 units with depth, the average abundance of AOB decreased 19-fold. The increase in pH may have decreased AOB growth and abundance.

To better understand the activity of the ammonia-oxidizing community in different sediment layers, potential ammonia oxidation rates were measured in the laboratory. Variations in the potential ammonia oxidation rates were not explained by the concentrations of *amoA* genes or mRNA in different sediment layers, as the rates did not exhibit any positive correlation with the concentrations of *amoA* or mRNA. Perhaps, the potential ammonia oxidation rates should be determined not only by the abundance and expression of AOB and AOA, but also by the phylotypes of AOB and AOA, as shown in Figures 6A,B, both of which consisted of different phylotype clusters, which may have different growth and nitrification rates (Bollmann et al., 2002, 2005; Tourna et al., 2011; Jong-Geol et al., 2012).

The results for the expression of *amoA* mRNA showed that the concentrations of AOB *amoA* mRNA was higher than that of AOA by 2.5- to 39.9-fold in the surface sediments (0–10 cm depth; Figure 4), although the copy numbers of the AOA *amoA* gene were higher than those of AOB by an order of magnitude in the surface sediments (0–6 cm depth; Figures 3B,C). The results indicated that ammonia oxidation was mainly carried out by AOB in surface sediments (0–10 cm depth), and that AOA might be the dominant ammonia-oxidizing microorganisms in deeper sediments (>10 cm depth), where only the AOA *amoA* gene was detected. A similar phenomenon was found in an agricultural soil, where AOB, rather than AOA, mainly conducted the ammonia oxidation, despite the fact that AOA *amoA* genes were more numerous than AOB *amoA* genes, and which was demonstrated by DNA-stable isotope probing (Jia and Ralf, 2009). In addition, in a temperate forest soil, it was also suggested that AOB are more involved than AOA in net nitrification in the top 5 cm of soil in July, and that AOA *amoA* genes are more numerous than AOB *amoA* genes in the topsoil (Onodera et al., 2010).

### Diversity of AOA and AOB

The diversity of AOB has been studied in various ecosystems with molecular tools, and it has been shown that AOB exhibit apparently high biodiversity in many aquatic ecosystems (Nicol et al., 2008; Wu et al., 2010; Jin et al., 2011; Sun et al., 2013). In this study, there were two AOB clusters: the *Nitrosospira* and *Nitrosomonas* clusters were found in sediments, and the latter was predominant in both sediment layers (Figure 6A). These results are consistent with previous studies of rhizoplanes of floating aquatic macrophytes, as well rice soils (Nicolaisen et al., 2004; Wang et al., 2009; Wei et al., 2011). *Nitrosomonas* were often detected in high-nitrogen environments, such as wastewater treatment plants (Geets et al., 2006; Stephanie et al., 2015), and some other studies have suggested that high concentrations of ${\text{NH}}_{4}^{+}-$N could enhance the development of *Nitrosomonas* spp. relative to *Nitrosospira* spp. (Bollmann et al., 2002, 2005).

Like AOB, the archaeal *amoA* gene was detected in different sediment layers, and all AOA fell within the *Nitrososphaera* and *Nitrosopumilus* clusters of the *Thaumarchaeota* phylum, with the latter being the dominant type (Figure 6B). A similar observation was found in the hyporheic zone of a eutrophic river (Wang et al., 2014b), where two distinct monophyletic clusters were also found, and the diversity of AOA decreased slightly with increasing sediment depth, but the *Nitrososphaera* cluster was the dominant cluster of archaeal ammonia oxidizers. In addition, the *Nitrososphaera* cluster represented the majority of AOA in many wastewater treatment plants, where *Nitrosopumilus* and *Nitrososphaera* clusters were jointly found (Limpiyakorn et al., 2013). The archaeal sequences were assigned into two branches with a clear difference between the surface and deeper samples, which may be attributed to the higher concentrations of TOC and ${\text{NH}}_{4}^{+}-$N in surface sediment. There was evidence that the *Nitrososphaera* cluster could bear higher amounts of TOC (Chen et al., 2008; Tourna et al., 2011; Liu et al., 2013) and ${\text{NH}}_{4}^{+}-$N (Tourna et al., 2011) than the *Nitrosopumilus* cluster. A similar phenomenon was also found in the sediments of the Dongjiang and Qiantang rivers (Liu et al., 2013; Sun et al., 2013). The AOA in deeper pond sediment were all grouped into the *Nitrosopumilus* cluster, which could be inhibited by organic carbon and prefer relatively lower carbon contents (Könneke et al., 2005).

In summary, our results showed that diversity of AOA and AOB decreased with increasing sediment depth and different dominant species were found at the different depths sampled. AOA were less active than AOB in surface sediments (0–10 cm depth) of the freshwater aquaculture pond, however, where AOA, as opposed to AOB, were the most abundant ammonia-oxidizing organisms. AOA might be the dominant ammonia-oxidizing microorganisms in deeper sediments, where only the AOA *amoA* gene was detected. This could be attributed to the fact that AOA are more resistant to low levels of DO. These results provide some useful information toward our understanding of freshwater pond sediment and their management, especially for the process of ammonia oxidation in fish pond sediment.

### Conflict of interest statement

The authors declare that the research was conducted in the absence of any commercial or financial relationships that could be construed as a potential conflict of interest.
